# Noninvasive Electrical Mapping Compared with the Paced QRS Complex for Optimizing CRT Programmed Settings and Predicting Multidimensional Response

**DOI:** 10.1007/s12265-023-10418-1

**Published:** 2023-09-06

**Authors:** Frances L. Morales, Derek J. Bivona, Mohamad Abdi, Rohit Malhotra, Oliver Monfredi, Andrew Darby, Pamela K. Mason, J. Michael Mangrum, Sula Mazimba, Robert W. Stadler, Frederick H. Epstein, Kenneth C. Bilchick, Pim J. A. Oomen

**Affiliations:** 1 University of Virginia Health System, Charlottesville, VA 22901 USA; 2 Medtronic plc., Mounds View, MN USA; 3 Department of Biomedical Engineeering, Edwards Lifesciences Foundation Cardiovascular Innovation and Research Center, University of California, Irvine, Irvine, CA USA

**Keywords:** Cardiac resynchronization therapy, Electrical mapping, Right ventricular function, Heart failure, Cardiac magnetic resonance

## Abstract

The aim was to test the hypothesis that left ventricular (LV) and right ventricular (RV) activation from body surface electrical mapping (CardioInsight 252-electrode vest, Medtronic) identifies optimal cardiac resynchronization therapy (CRT) pacing strategies and outcomes in 30 patients. The LV80, RV80, and BIV80 were defined as the times to 80% LV, RV, or biventricular electrical activation. Smaller differences in the LV80 and RV80 (|LV80-RV80|) with synchronized LV pacing predicted better LV function post-CRT (*p* = 0.0004) than the LV-paced QRS duration (*p* = 0.32). Likewise, a lower RV80 was associated with a better pre-CRT RV ejection fraction by CMR (*r* =  − 0.40, *p* = 0.04) and predicted post-CRT improvements in myocardial oxygen uptake (*p* = 0.01) better than the biventricular-paced QRS (*p* = 0.38), while a lower LV80 with BIV pacing predicted lower post-CRT B-type natriuretic peptide (BNP) (*p* = 0.02). RV pacing improved LV function with smaller |LV80-RV80| (*p* = 0.009). In conclusion, 3-D electrical mapping predicted favorable post-CRT outcomes and informed effective pacing strategies.

**Graphical Abstract:**

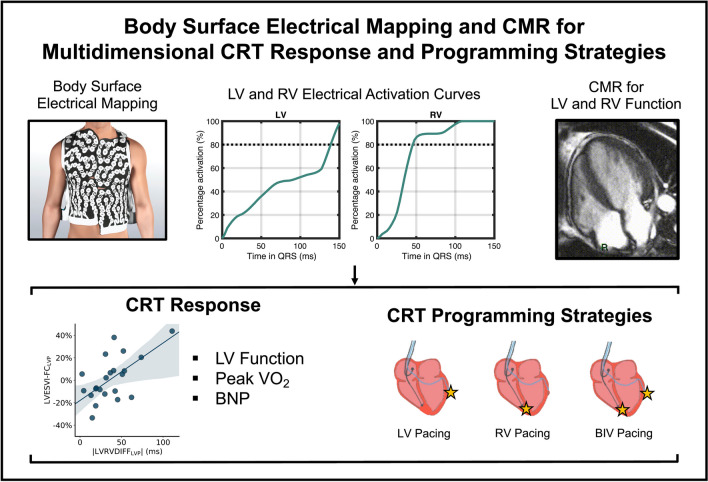

**Supplementary Information:**

The online version contains supplementary material available at 10.1007/s12265-023-10418-1.

## Introduction

Cardiac resynchronization therapy (CRT) can be a life-saving intervention for many patients with heart failure [1–3] and is associated with improved ventricular function and other response measures [4, 5], albeit with a significant nonresponse rate associated with suboptimal patient selection, lead placement, and other factors [6–8]. Furthermore, CRT response is complex, and parameters such as peak oxygen consumption [3] and B-type natriuretic peptides have been shown to be important prognostic indicators after CRT [9] and in heart failure [10], in general, particularly when formulated as a multidimensional indicator of CRT response [11]. Differences in electrical and mechanical activation of the left and right ventricles (LV and RV) among patients with heart failure referred for CRT provide the justification for personalized approaches to implementation and patient selection strategies for CRT [7], as optimal synchrony with CRT has been strongly associated with improved clinical outcomes, exercise capacity, and neurohormonal profiles [8, 11–13].

Current guidelines recommend using the QRS duration (QRSd) and the type of bundle branch block *before* CRT to guide patient selection [14], and the paced QRSd *after* CRT is considered a valuable predictor of long-term response [15]. A known limitation of this approach is that the QRSd and type of bundle branch block before CRT provide only a crude measure of LV- and RV-specific activation times. In this regard, the possibility of right bundle branch block (RBBB) masquerading as left bundle branch block (LBBB) has been known for many decades [16].

In this present study, we provide a novel approach for the study of electrical activation in CRT using three-dimensional electrical body surface mapping [13, 17] to address this clinical problem. In particular, as rates of RV and LV electrical activation may be variable throughout the QRS duration, we hypothesized that robust parameters of RV and LV activation—the time to 80% of RV activation (RV80) and the time to 80% of LV activation (LV80)—would provide more accurate predictions for response to therapy than the crude QRS duration after CRT pacing. In other words, the rationale for these parameters is that the proportion of RV, LV, or biventricular (BIV) chamber activation does not consistently correspond to the proportion of the QRS completed at any given time point between the start and the end of the QRS. The study builds on prior studies of body surface electrical mapping studies [17–21] in the following ways: (1) characterizing the consistency of electrical activation in the RV and LV over time; (2) evaluating correlations with cardiac MRI; and (3) demonstrating the utility of RV80 and LV80 assessments before and after CRT to inform optimal CRT programming strategies and predict multidimensional measures of CRT response related to LV function, peak oxygen consumption with exercise, and neurohormonal activation.

## Methods

### Study Design

All procedures followed were in accordance with the ethical standards of the responsible committee on human experimentation (institutional and national) and with the Helsinki Declaration of 1975, as revised in 2000 (5). Informed consent was obtained from all patients for being included in the study. This prospective cohort study was approved by the Institutional Review Board for Human Subjects Research at the University of Virginia. The cohort included 30 adult patients aged 25 to 89 years old with an indication for a de novo CRT device or an upgrade to a CRT device from an existing pacemaker or ICD. All participants were required to have chronic systolic heart failure, left ventricular ejection fraction (LVEF) 35% or less, and a guideline-based class I or II indication for CRT. Exclusion criteria included inability to provide informed consent, pregnancy, metal implants, cerebral aneurysm clips, cochlear implants, other metallic implants contraindicated with MRI, severe claustrophobia, acute kidney injury, acute renal failure or chronic kidney disease with GFR less than 45 cc/minute, history of liver transplant, and gadolinium allergy.

### Pre-CRT CMR and Echocardiography

Prior to CRT, patients had echocardiography and cardiac magnetic resonance (CMR) for baseline data on global cardiac function, left ventricular volumes, and myocardial substrate. The research CMR protocol included steady-state free precession cine imaging to obtain ventricular geometries and cavity volumes, cine DENSE imaging to determine circumferential strain for characterization of mechanical dyssynchrony [22, 23], and late gadolinium enhancement for direct visualization of myocardial scar. CURE-SVD as a robust dyssynchrony parameter derived from DENSE strain was determined as previously described [8, 12]. Parameters for both RV and LV function were obtained. Standard 2D echocardiographic images were obtained to compute the baseline left ventricular end-diastolic volume index (LVEDVI), left ventricular end-systolic volume index (LVESVI), and the LVEF using Simpson’s rule for 2- and 4-chamber long-axis views.

### CardioInsight Mapping

Noninvasive electro-anatomical mapping of electrical activation timing was performed with CardioInsight body surface mapping of electrocardiographic signals during the CRT procedure and 6 months later. The CardioInsight system uses a 252-electrode vest to map electrocardiographic signals recorded on the chest onto the epicardial surface of the heart based on the contours defined by a cardiac CT scan recorded prior to the CRT procedure. During both the baseline and 6-month follow-up visits, CardioInsight mapping was performed at the following pacing settings: atrial-only pacing, atrioventricular sequential pacing using only the RV lead for ventricular pacing (RVP), atrioventricular pacing using only the LV lead for ventricular pacing (LVP), atrioventricular pacing with simultaneous biventricular pacing (BIVP), and BIVP with an LV-first offset of 30 ms. Atrioventricular timing during CRT pacing was determined either with a device-based algorithm or electrocardiogram (ECG) optimization.

### Post-CRT Echocardiography

The standard 2D echocardiography protocol performed prior to CRT was performed 6 months after CRT with imaging sets from four different pacing settings corresponding to the CardioInsight electrical mapping pacing configurations during electrical 3D mapping. Specifically, the imaging sets were performed with atrial-only pacing, atrioventricular sequential pacing with RVP, atrioventricular pacing with LVP, atrioventricular pacing with simultaneous BIVP, and BIVP with an LV-first offset of 30 ms. LVESVI fractional change (LVESVI-FC) was defined as the (LVESVI_POST-CRT_ − LVESVI_PRE-CRT_)/LVESVI_PRE-CRT_.

### Novel Cardiac Electrical Activation Parameters: LV80, RV80, and BIV80

The 3D electrical maps derived from CardioInsight were used to calculate the total percentage of the epicardial surface that was activated during the R-R interval at the patient’s intrinsic rhythm and at various pacing settings. The novel parameters LV80 and RV80 were defined as the time on the R-R interval at which 80% of the left and right ventricular epicardial surfaces of the heart were electrically activated. Similarly, BIV80 was defined as the time at which 80% of both ventricles were electrically activated. In contrast to parameters derived from a standard 12-lead ECG such as QRS duration, the novel LV80 and RV80 parameters can distinguish between changes in LV and RV electrical function.

### Assessment of Variable Temporal RV/LV Activation

In order to assess variable temporal activation of the RV and LV, plots of the RV80 and LV80 versus QRS duration were obtained. The slopes of regression lines for LBBB and RBBB patients are approximations of the average LV80/QRS, RV80/QRS, and BIV80/QRS in LBBB and RBBB. With constant rates of RV and LV activation throughout the QRS duration (linear activation versus time functions), 80% of RV myocardial surface area would be expected to have activation within the RV80 time. As 100% of the RV myocardial surface is assumed to be activated within the QRS duration, the slope of the RV80/QRS regression line would be expected to be 0.8 with linear RV electrical activation over time. The same reasoning applies to the LV80/QRS and BIV80/QRS lines. Deviation from 0.8 can then be interpreted as electrical activation that is variable over time.

### Statistical Analysis

Statistical tests were first performed to identify any differences in baseline characteristics and 6-month CRT response measures between patients with LBBB and patients with RBBB. Shapiro-Wilk tests were used to assess normality of continuous variables (Supplemental Table 1). Kruskal-Wallis tests were used for comparisons of continuous variables in LBBB and RBBB groups, and the Fisher exact tests were used to compare discrete variables between groups. One-sample *t*-tests were used to determine whether the ratios of the time to 80% RV, LV, or total ventricular activation to the QRS duration (RV80/QRS, LV80/QRS, and BIV80/QRS) were different than the default value of 0.8 in a particular grouping of patients. In other words, the null hypothesis for this test was that a particular ratio of electrical parameters was 0.8, and the alternative hypothesis was that the parameter was different from 0.8. The RV80/QRS, LV80/QRS, and BIV80/QRS were then regressed on LBBB v. RBBB status, and interaction terms were added to evaluate the influence of scar, ischemic etiology of cardiomyopathy, the QRS-LV electrogram (Q-LV) time, and QRS duration influences this relationship. In order to assess the relationship between the electrical activation parameters and multidimensional CRT response parameters (LVESV-FC, post-CRT BNP, and peak VO_2_), robust linear regression was used. All statistical analyses were performed using R and the *statsmodels* package in Python.

## Results

### Baseline Characteristics and Response Measures of Patient Cohort

The baseline characteristics for the 30 patients (median age 68.0 with interquartile range [IQR] 56.3 to 72.5; 36.7% female) are shown in Table 1. Patients were dichotomized into two groups: LBBB v. RBBB; 20 patients (66.7%) had LBBB while 10 patients (33.3%) had RBBB. Patients with RBBB had a longer QRS duration (median 162.5 ms with IQR 160.0 to 173.5 ms v. median 150.5 ms with IQR 139.5 to 161.5 ms; *p* = 0.021) and lower pre-CRT peak VO_2_ (median 12.6 mL/kg/min with IQR 10.8 to 14.3 mL/kg/min v. 14.4 mL/kg/min with IQR 14.4 to 15.8 mL/kg/min; *p* = 0.017) compared with LBBB patients. With respect to CRT response measures, the median LVESVI-FC for the entire cohort was −0.14 (IQR −0.26 to −0.0064); the median log-transformed post-CRT BNP level was 5.3 (IQR 3.9 to 5.9); and the median change in peak VO_2_ was 0 mL/kg/min (IQR −0.18 to 0.45 mL/kg/min). Although there were no significant differences in the fractional change in LVESVI (LVESVI-FC) in LBBB v. RBBB (*p* = 0.28) or log of the post-CRT BNP in LBBB v. RBBB (*p* = 0.23), there was more favorable coupling of the peak VO_2_ to the LVESVI-FC in RBBB v. LBBB (*p* = 0.0031).

**Table 1:** Baseline characteristics and response outcomes by bundle branch block morphology

	All (*N* = 30)	LBBB (*N* = 20)	RBBB (*N* = 10)	*p* value
Demographics				
Age, years	68.0 (56.3–72.5)	67.0 (56.0–70.3)	71.5 (61.3–78.0)	0.070
BMI	30.6 (25.5–36.0)	31.2 (25.3–37.0)	29.4 (25.8–32.2)	0.32
Weight, kg	93.3 (77.2–102.9)	92.1 (74.4–101.1)	93.3 (84.2–115.2)	0.25
Female	11 (36.7)	10 (50.0)	1 (10.0)	0.048*
NYHA heart failure class				0.70
II	19 (63.3)	12 (60.0)	7 (70.0)	
III	11 (36.7)	8 (40.0)	3 (30.0)	
IV	0 (0.0)	0 (0.0)	0 (0.0)	
Race				0.53
Black	3 (10.0)	3 (15.0)	0 (0.0)	
White/Other	27 (90.0)	17 (85.0)	10 (100.0)	
SHFMM	0.28 (0.10–0.47)	0.28 (0.0035–0.36)	0.24 (0.13–0.60)	0.46
Comorbid conditions				
Ischemic cardiomyopathy	10 (33.3)	5 (25.0)	5 (50.0)	0.23
Hypertension	18 (60.0)	12 (60.0)	6 (60.0)	1
Atrial fibrillation	8 (26.7)	4 (20.0)	4 (40.0)	0.38
Chronic kidney disease	14 (46.7)	8 (40.0)	6 (60.0)	0.44
Diabetes mellitus	18 (60.0)	11 (55.0)	7 (70.0)	0.69
Prior CABG	9 (30.0)	6 (30.0)	3 (30.0)	1
Medications				
Beta-blocker	27 (90.0)	18 (90.0)	9 (90.0)	1
ACE inhibitor or ARB	26 (86.7)	17 (85.0)	9 (90.0)	1
Loop diuretic	17 (56.7)	11 (55.0)	6 (60.0)	1
Digoxin	1 (3.3)	1 (5.0)	0 (0.0)	1
Statin	18 (60.0)	11 (55.0)	7 (70.0)	0.69
Laboratory studies, vital signs, & exercise testing				
Systolic BP, mm Hg	114.0 (107.3–128.0)	117.5 (107.8–128.0)	110.0 (102.5–129.0)	0.28
Sodium, mEq/L	137.0 (136.0–139.8)	137.0 (135.5–140.0)	138.0 (136.0–139.0)	0.38
Creatinine, mg/dL	1.2 (0.9–1.4)	1.2 (0.9–1.4)	1.2 (0.85–1.3)	0.48
Hemoglobin, g/dL	13.0 (11.9–14.3)	12.9 (11.6–14.0)	13.5 (12.9–15.3)	0.12
GFR, mL/min/1.72m^2^	62.0 (51.0–79.5)	62.0 (51.0–79.5)	62.5 (50.8–78.3)	0.5
Log(BNP)	5.6 (4.9–6.2)	5.6 (4.8–5.6)	5.9 (5.4–6.3)	0.076
Peak VO_2_, mL/kg/min	14.4 (12.3–15.4)	14.4 (14.4–15.8)	12.6 (10.8–14.3)	0.017*
CMR & echocardiography assessment parameters				
LVEF, %	23.9 (16.6–32.8)	23.9 (18.3–35.5)	21.9 (16.1–26.8)	0.20
LVEDVI, mL/m^2^	108.5 (94.2–155.3)	108.5 (90.9–151.8)	109.3 (98.1–155.3)	0.39
LVESVI, mL/m^2^	86.3 (62.9–114.5)	86.5 (60.0–113.5)	82.8 (68.2–128.8)	0.48
RVEF, %	34.5 (30.2–39.7)	35.0 (32.7–40.2)	31.9 (19.8–34.6)	0.073
RVEDVI, mL/m^2^	66.5 (53.1–85.3)	66.5 (51.2–85.0)	71.0 (56.5–90.0)	0.28
RVESVI, mL/m^2^	37.1 (31.7–53.6)	37.1 (30.3–43.4)	43.4 (36.4–73.8)	0.13
LGE presence	10.0 (33.3)	5 (25.0)	5 (50.0)	0.23
CURE-SVD	0.64 (0.52–0.73)	0.64 (0.47–0.71)	0.63 (0.54–0.73)	0.41
Electrical parameters				
QRS, ms	160.0 (141.3–165.3)	150.5 (139.5–161.5)	162.5 (160.0–173.5)	0.021*
QLV, ms	100.0 (84.5–119.8)	111.5 (89.3–120.0)	91.6 (80.9–100.0)	0.14
Response measures at 6-months post-CRT				
Fractional change in LVESVI	−0.14 (−0.26 to −0.0064)	−0.18 (−0.25 to −0.033)	−0.10 (−0.28–0.03)	0.28
Log(BNP)	5.3 (3.9–5.9)	5.0 (3.9–5.6)	5.3 (4.4–6.1)	0.23
Change in peak VO_2_, mL/kg/min	0.0 (−0.18–0.45)	−0.001 (−0.28–0.016)	0.85 (035–2.1)	0.0031*

*ACE* angiotensin-converting enzyme, *ARB* angiotensin receptor blocker, *BMI* body mass index, *BNP* B-type natriuretic peptide, *BP* blood pressure, *CABG* coronary artery bypass graft, *CURE-SVD* circumferential uniformity ratio estimate with singular value decomposition, *GFR* glomerular filtration rate, *LBBB* left bundle branch block, *LGE* late gadolinium enhancement, *LVEDVI* left ventricular end-diastolic volume index, *LVEF* left ventricular ejection fraction, *LVESVI* left ventricular end-systolic volume index, *NYHA* New York Heart Association, *QLV* QRS-LV electrogram time, *RBBB* right bundle branch block, *RVEDVI* right ventricular end-diastolic volume index, *RVEF* right ventricular ejection fraction, *RVESVI* right ventricular end-systolic volume index, *SHFM* Seattle Heart Failure Model

### Variable Rates of Ventricular Activation

**Fig. 1:**
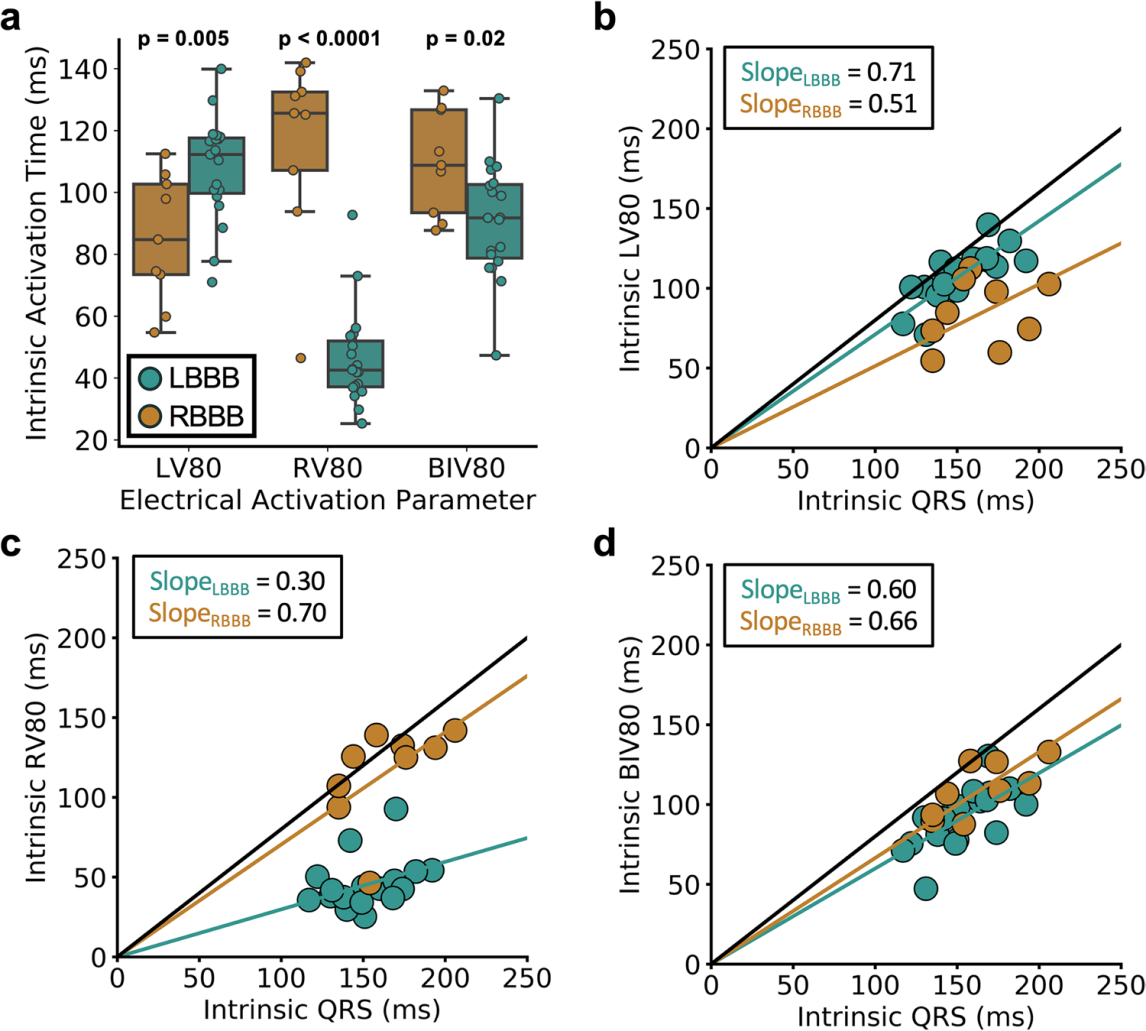
CRT electrical activation parameters. **a** Differences in intrinsic RV80, LV80, and BIV80 are shown for LBBB and RBBB. **b**-**d** Variability in electrical activation based on LBBB and RBBB are shown for **b** LV80, **c** RV80, and **d** BIV80, and the slope for each relationship for each case, such that a difference is slopes, indicates a non-constant rate of electrical activation throughout the QRS duration

Overall, the LV80 with native conduction (“intrinsic LV80”) was significantly greater in LBBB patients versus RBBB patients (*p* = 0.005) whereas the RV80 with native conduction (“intrinsic RV80”, *p* < 0.0001) and BIV80 with native conduction (“intrinsic BIV80”, *p* = 0.02) were significantly greater in RBBB patients than in LBBB patients (Fig. 1a).

Inspection of LV80 versus QRS (Fig. 1b), RV80 versus QRS (Fig. 1c), and BIV80 versus QRS (Fig. 1d) scatter plots demonstrate variable rates of RV and LV free wall activation. Slopes of the electrical activation versus time functions that deviate from 0.8 indicate non-constant ventricular electrical activation; in other words, if the proportion of ventricular free wall myocardium activated during the first 80% of the QRS duration is significantly different from 80%, then the rates of myocardial surface area activation are different during the initial 80% and final 20% of the QRS duration.

As shown in Table 2, the slopes for baseline LV80 v. baseline QRS in LBBB and RBBB patients were 0.71 and 0.5 (*p*<0.0001 for comparison), respectively, and the slopes for baseline RV80 v. baseline QRS were 0.30 and 0.70 for LBBB and RBBB patients, respectively (*p*<0.0001 for comparison). One-sample *t*-tests compared to the default value of 0.8 (corresponding to a constant rate of electrical activation throughout the QRS duration) showed significant differences from 0.8 for the LV80/QRS in both LBBB and RBBB patients (*p*=0.0002 for both) and the RV80/QRS in LBBB patients (*p*<0.0001). Based on linear regression models for the outcomes of RV80/QRS and LV80/QRS with interaction terms, the differences in the RV80/QRS in LBBB v. RBBB and LV80/QRS in LBBB v. RBBB did not depend on the presence of late gadolinium enhancement, ischemic etiology of cardiomyopathy, QRS duration, or the QRS-LV electrogram time (QLV) (*p* value for interaction terms > 0.1 in all models).

**Table 2:** Ratio parameters for time to 80% ventricular chamber activation/QRS without CRT pacing (native conduction)

Electrical parameter	Patient subgroup	CRT status	Parameter calue	*p* value v. default value of 0.8	*p* value v. RBBB patients*	*p* value for LBBB*QRSd interaction term**
LV80/QRS	LBBB	Off	0.71	0.0002	<0.00001	0.17
LV80/QRS	RBBB	Off	0.50	0.0002	.	.
RV80/QRS	LBBB	Off	0.30	<0.0001	<0.00001	0.50
RV80/QRS	RBBB	Off	0.70	0.10	.	.
BIV80/QRS	LBBB	Off	0.60	0.10	0.0504	0.03
BIV80/QRS	RBBB	Off	0.66	0.20	.	.

*Corresponds to linear model LV80/QRS~LBBB, RV80/QRS~LBBB, or BIV80/QRS~LBBB, where LBBB=1 or 0

**Corresponds to linear model LV80/QRS~LBBB*QRS, RV80/QRS~LBBB*QRS, or BIV80/QRS~LBBB*QRS, where LBBB=1 or 0 and QRS is the QRS duration

The BIV80/QRS for LBBB and RBBB were 0.60 and 0.66, respectively (*p*=0.0504 for comparison between LBBB and RBBB). In addition, neither LBBB patients nor RBBB patients had a BIV80/QRS that was significantly different from 0.8, indicating overall constant biventricular activation despite non-constant LV and RV activation, which is likely related to some cancellation of the variability of RV and LV activation functions when RV and LV activation are parameterized together as the BIV80. Although the difference in BIV80/QRS was not significantly different in LBBB and RBBB based on the *p* value of 0.0504, the BIV80/QRS was significantly different in LBBB and RBBB after adjustment for the QRS duration (*p*=0.02 for the LBBB/RBBB covariate). The *p* value for the interaction term LBBB*QRS was 0.03 with a positive coefficient, indicating that the BIV80/QRS is more likely to be different between LBBB and RBBB with longer QRS durations.

**Fig. 2:**
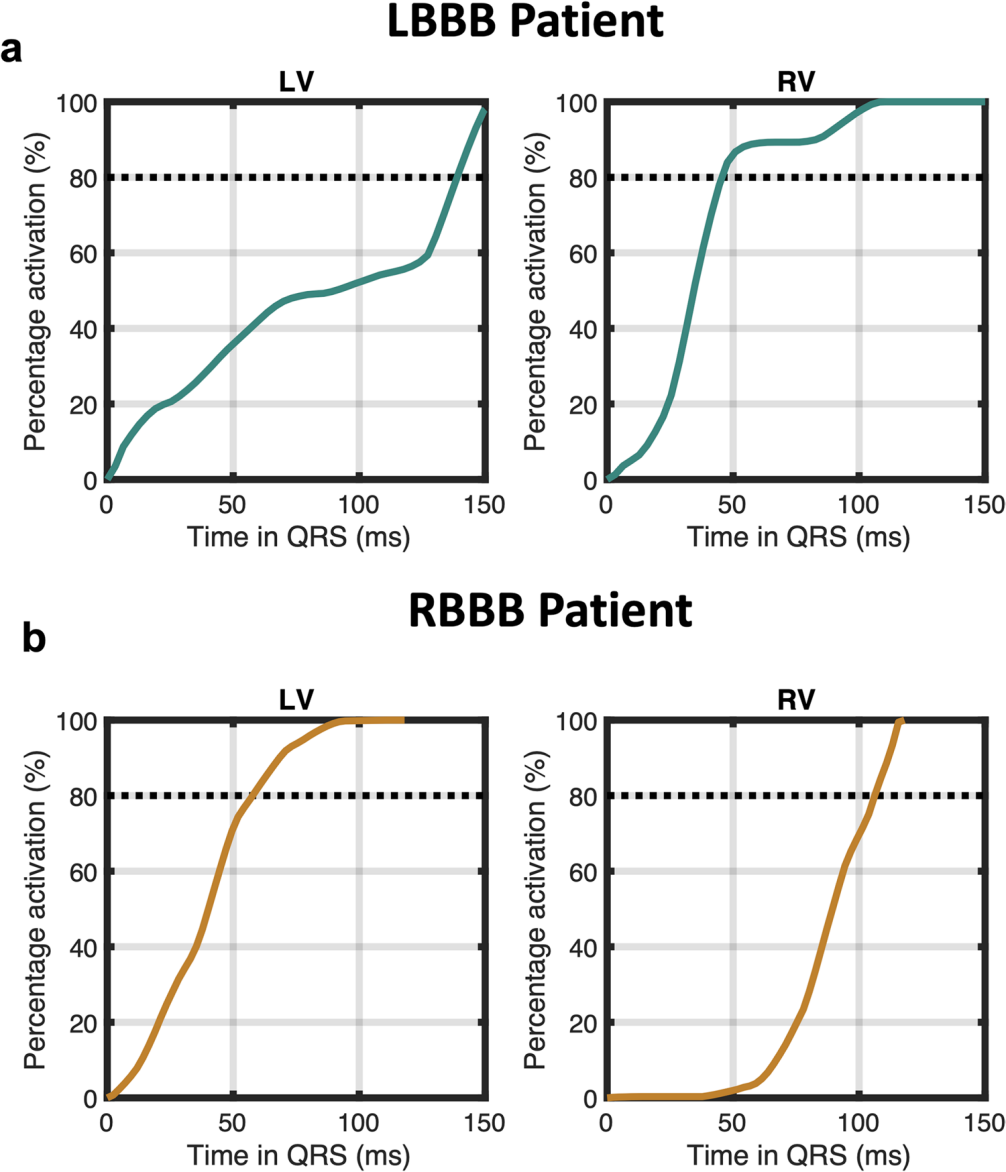
Sample variable electrical activation curves. Typical electrical activation curves demonstrating variability of activation are shown for patients with LBBB (**a**) and RBBB (**b**). The plots for the proportion of electrical activation versus time are not linear, which demonstrates non-constant electrical activation through the QRS duration

Examples of variable electrical activation versus time functions for the LV and RV are shown in Fig. 2 for a patient with LBBB and a patient with RBBB. In the patient with LBBB, 80% of RV activation occurs in 50 ms, and the remaining RV activation occurs in the remaining 80 ms, which gives an RV80/QRS of 0.38 (well less than the expected 0.80 under conditions of linear activation over time). In the RBBB patient, there is more variability in the LV80/QRS compared with the RV80/QRS, with 80% activation of the LV occurring within 60 ms and the remaining 20% in the last 70 ms (RV80/QRS = 0.46). This is consistent with the observations in Fig. 1 regarding more variability in the LV activation versus time functions for RBBB patients and more variability in the RV activation versus time functions in LBBB patients.

### Impact of Pacing on the Consistency of LV and RV Activation Rates

**Fig. 3:**
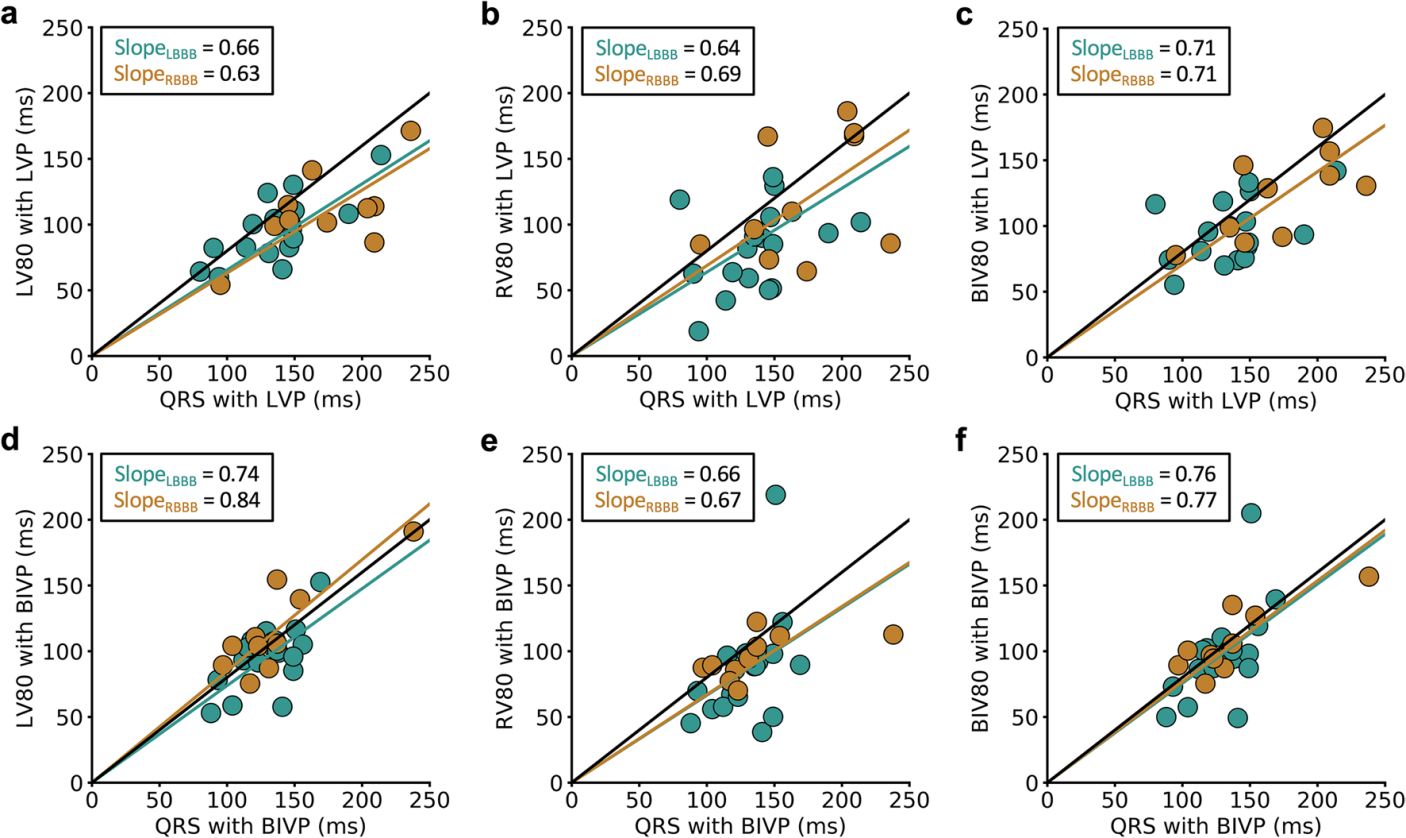
Effects of pacing on linearity of electrical activation for the RV and LV. **a** LV80, **b** RV80, and **c** BIV80 are plotted versus QRS duration for LV pacing. Similar plots are shown for BIV pacing in **d–f**. Compared with Fig. 1, the slopes for RBBB and LBBB are more consistent with LV pacing and BIV pacing

Figure 3 demonstrates that CRT implemented either with synchronized LV pacing (LVP) or biventricular pacing (BIVP) makes RV and LV electrical activation rates more consistent over time in both RBBB and LBBB. In addition, biventricular pacing results in more consistent left ventricular activation than LVP in RBBB with LV80/QRS increasing from 0.63 to 0.84 (*p* = 0.004); however, the difference LV80/QRS between pacing strategies was not different in LBBB (0.66 v. 0.74; *p* = 0.4).

### Differences in Electrical Activation Parameters Among Dyssynchrony Type and Pace Settings

**Fig. 4:**
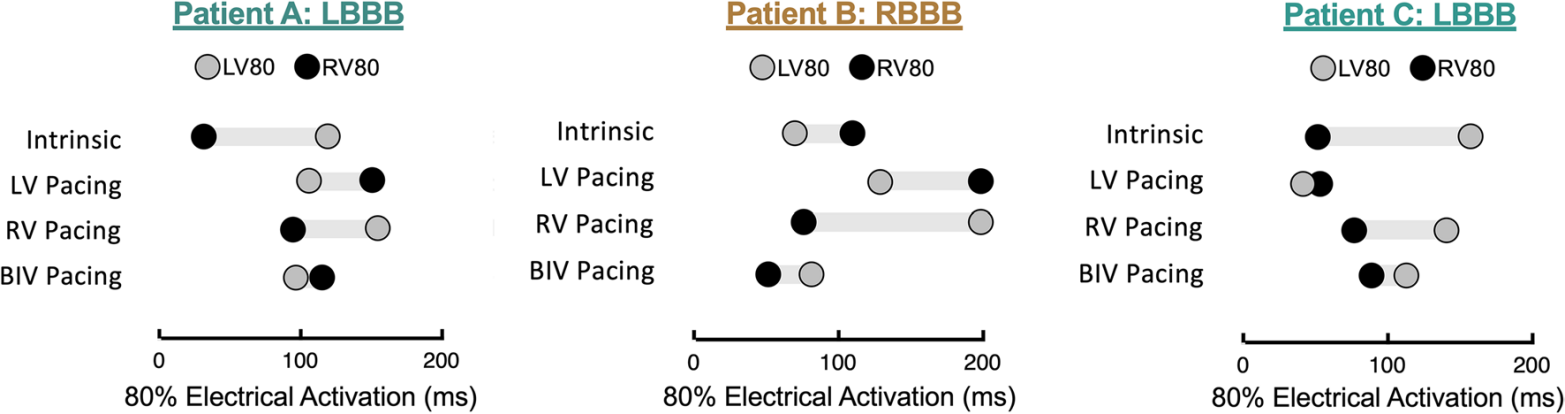
Pacing configuration plots. Novel pacing configuration plots are shown for easy visualization of electrical activation of the RV and LV for different pacing modes, such as BIVP and LVP. These plots demonstrate the extent to which the RV80 and LV80 shorten or lengthen relative to the intrinsic rhythm and the similarity between the RV80 and LV80 with each mode, as a measure of electrical interventricular synchrony

The distributions of the LV80, RV80, and BIV80 electrical activation parameters for representative patients with LBBB and RBBB are shown in the “pacing configuration plots” in Fig. 4. These novel pacing configuration plots help with rapid visualization of the effect of pacing settings on electrical synchrony. In the LBBB patient (Fig. 4a), the LV80 is larger than the RV80 intrinsically. When the pacing setting is programmed to LV pacing, the LV80 decreases as a response to the LV pacing, and the RV80 increases. During BIVP, the LV80 decreases and RV80 increases with respect to the intrinsic rhythm, and the difference between these measures is the smallest compared to any setting. In this patient, the time it takes for 80% of LV and 80% of the RV to be electrically activated is nearly the same. Pacing configuration plots for additional patients are shown in Fig. 4b (a patient with RBBB) and Fig. 4c (a patient with LBBB). These plots suggest that BIVP results in the optimal electrical activation for patients A and B, yet the plot for patient C suggests that LVP is optimal. Differences in LV80 and RV80 along with BIV80 for each pace setting were compared among LBBB and RBBB patients and are shown in Supplemental Fig. 1. Only the RV80 with RV pacing (*p* = 0.03) was significant among the two groups. Supplemental Fig. 1 also shows the distributions of the LVEDVI, LVESVI, and LVEF measures derived from echocardiography for each pace setting among LBBB and RBBB patients.

### Predictions of Echo-Derived Volumes and CRT Response Measures with Electrical Activation

**Fig. 5:**
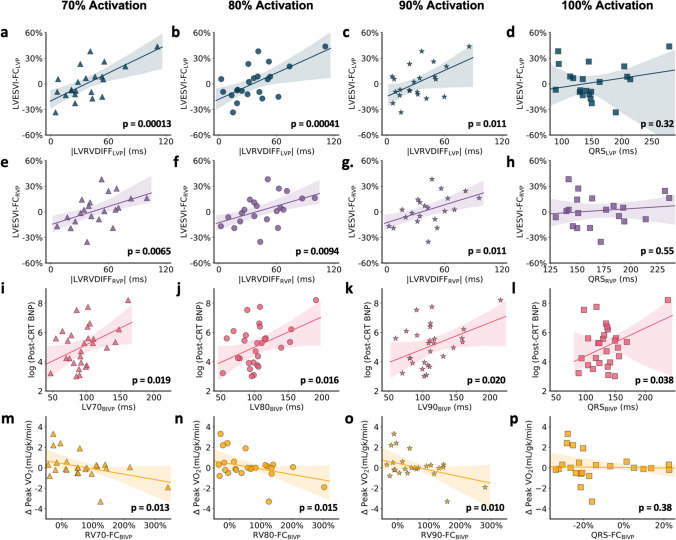
Associations of electrical chamber-specific baseline parameters with CRT outcomes. The fractional change in the LVESVI (multiplied by 100 to give a percentage) with LVP is plotted versus the absolute difference between LV80 and RV80 with LVP in **b**, and the comparison with QRS duration is provided in **d**. Corresponding plots for 70% and 90% activation are shown in **a** and **c**, respectively. The fractional change in the LVESVI with RVP is plotted versus the absolute difference between the LV80 and RV80 with RVP in **f**, and the comparison with QRS duration is provided in **h**. Corresponding plots for 70% and 90% activation are shown in **e** and **g**, respectively. The log of the post-CRT BNP is plotted versus the LV80 with biventricular pacing in **j**, and the comparison with QRS duration is provided in **l**. Corresponding plots for 70% and 90% activation are shown in **i** and **k**, respectively. The peak VO_2_ is plotted versus the fractional change in the RV80 (also multiplied by 100 to give a percentage) with **n** biventricular pacing, and the comparison with QRS duration is provided in **p**. Corresponding plots for 70% and 90% activation are shown in **m** and **o**, respectively

We used robust linear regression to assess relationships between chamber-specific electrical parameters and CRT response measures, then compared them with the performance of the paced QRS duration (QRSd). Figure 5b shows that a lower absolute value of the difference of LV80 and RV80 activation times (|LVRVDIFF|, an indicator of interventricular electrical synchrony), during LVP, was associated with better LV function with LVP compared with baseline (LVESVI-FC_LVP_; *p* = 0.0004); the |LVRVDIFF| performed better than the paced QRSd for this prediction scenario, as the QRSd with LVP was not significantly associated with LVESVI-FC_LVP_ (*p* = 0.38) **(**Fig. 5d**)**.

An analogous result was obtained with |LVRVDIFF| during RVP, as a lower |LVRVDIFF| during RVP was associated with better LV function with RVP compared to baseline (LVESVI-FC_RVP_) function was better with RV pacing (Fig. 5f) (*p* = 0.0094), once more highlighting the importance of better electrical interventricular synchrony. Of note, the QRSd with RVP was not significantly associated with the LVESVI-FC during RVP **(**Fig. 5h**)** (*p* = 0.55).

Lower LV80 was associated with lower post-CRT BNP (Fig. 5j) (*p* = 0.016), and this relationship was more apparent than when the paced QRSd was used (*p* = 0.038) (Fig. 5l). In addition, greater improvements in peak VO_2_ were present when the RV80 fractional change decreased with biventricular pacing, where RV80-FC = (RV80_BIVP_ − RV80_PRE-CRT_)/RV80_PRE-CRT_ < 100% (Fig. 5n), while the change in peak VO_2_ with BIVP was not associated with the fractional change in QRSd with biventricular pacing, which was calculated in a similar fashion (Fig. 5p). Since an RV activation parameter was evaluated in this model, a model adjusted by the LV lead location as also evaluated in this model (Δ peak VO_2_ ~ RV80-FC_BIVP_ + LV_Lead_Position). Although there was no significant interaction, both covariates were significant predictors in the model (*p*=0.026 and *p*=0.018, respectively), which had an overall *R*^2^ of 0.41 (model *p*=0.0028).

Figure 5 also shows that electrical activation parameters evaluated at 70-80% of the QRS complex had greater associations with electrical activation parameters calculated at 90–100% of the QRS complex. This can be seen by inspecting the plots and *p* values for the first two columns of Fig. 5 compared with the last two columns of Fig. 5. For example, the *p* values for robust regression of LVESVI-FC_LVP_ on LV70-RV70-DIFF_LVP_ and LVESVI-FC_LVP_ on LV80-RV80-DIFF_LVP_ are on the order 10^−4^ (0.00013 to 0.00041), while the *p* values for LVESVI-FC_LVP_ on LV90-RV90-DIFF_LVP_ is on the order of 10^−2^ (0.011), and the *p* value using just the full QRS is not significant. This demonstrates that electrical parameters at 70–80% of the QRS duration had the strongest relationship with response. Progressively weaker relationships with response were observed with the use of electrical activation parameters based on 90% and 100% of the QRS complexes.

The impact of electrical activation timing at the LV pacing site for each model in Fig. 5 is demonstrated in Table 3, which shows the models adjusted for either the QRS-LV-electrogram time (Q-LV) or the ratio of the Q-LV/QRS as measures of whether the LV pacing leads were implanted in areas of late activation with determination of the *R*^2^ statistic, *p* value of the model, and Pearson’ correlation coefficients for models with a single covariate. This demonstrates that the degree of late activation at the LV pacing site had some additional predictive value to the electrical activation parameter from 3-D mapping in some of the models. With respect to other electrical parameters, the baseline PR interval did not improve the models below. Of note, the A-V pacing delays in this study were selected based on either the device-recommended A-V delay or the A-V delay resulting in the most narrow QRS complex, as is typically performed in clinical practice.

**Table 3:** Linear models for CRT outcome parameters including 3-D mapping electrical parameters and parameters of electrical activation at the LV pacing site

Linear model structure: LVESVI-FC_LVP_ ~ LV80-RV80-DIFF_LVP_ (+ Q-LV or Q-LV/QRS)			
Covariates	*R*^2^ (*p* value)	Corr.	Notes
LV80-RV80-DIFF_LVP_	0.36 (0.0033)	*r* = 0.60	
LV80-RV80-DIFF_LVP_+Q-LV	0.36 (0.017)		*p*=0.59 for Q-LV covariate
Q-LV	0.07 (0.26)	*r* = 0.26	
LV80-RV80-DIFF_LVP_+Q-LV/QRS	0.35 (0.021)		*p*=0.021 for Q-LV/QRS covariate
Q-LV/QRS	0.01 (0.67)	*r* = 0.10	
Linear model structure: LVESVI-FC_RVP_ ~ LV80-RV80-DIFF_RVP_ (+ Q-LV or Q-LV/QRS)			
Covariates	*R*^2^(*p* value)	Corr.	Notes
LV80-RV80-DIFF_RVP_	0.22 (0.030)	*r* = 0.47	
LV80-RV80-DIFF_RVP_+Q-LV	0.40 (0.013)		*p*=0.020: Q-LV covariate
Q-LV	0.29 (0.014)	*r* = 0.54	
LV80-RV80-DIFF_RVP_+Q-LV/QRS	0.39 (0.016)		*p*=0.030: Q-LV/QRS covariate
Q-LV/QRS	0.26 (0.67)	*r* = 0.51	
Linear model structure: Log (Post-CRT BNP) ~ LV80_BIVP_ (+ Q-LV or Q-LV/QRS)			
Covariates	*R*^2^ (*p* value)	Corr.	Notes
LV80_BIVP_	0.20 (0.018)	*r* = 0.44	
LV80_BIVP_+Q-LV	0.24 (0.042)		*p*=0.77: Q-LV covariate
Q-LV	0.002 (0.82)	*r* = 0.05	
LV80_BIVP_ +Q-LV/QRS	0.39 (0.016)		*p*=0.030: Q-LV/QRS covariate
Q-LV/QRS	6×10^−9^ (0.99)	*r* = 0.00008	
Linear model structure: Δ peak VO_2_ ~ RV80-FC_BIVP_ (+ Q-LV or Q-LV/QRS)			
Covariates	*R*^2^ (*p* value)	Corr.	Notes
RV80-FC_BIVP_	0.24 (0.013)	*r* = 0.49	
RV80-FC_BIVP_+Q-LV	0.36 (0.0075)		*p*=0.056: Q-LV covariate
Q-LV	0.15 (0.081)	*r* = 0.29	
RV80-FC_BIVP_+Q-LV/QRS	0.33 (0.012)		*p*=0.098: Q-LV/QRS covariate
Q-LV/QRS	0.10 (0.11)	*r* = 0.31	

### Association of Decreased RV Function with Variable RV Electrical Activation Over Time

**Fig. 6:**
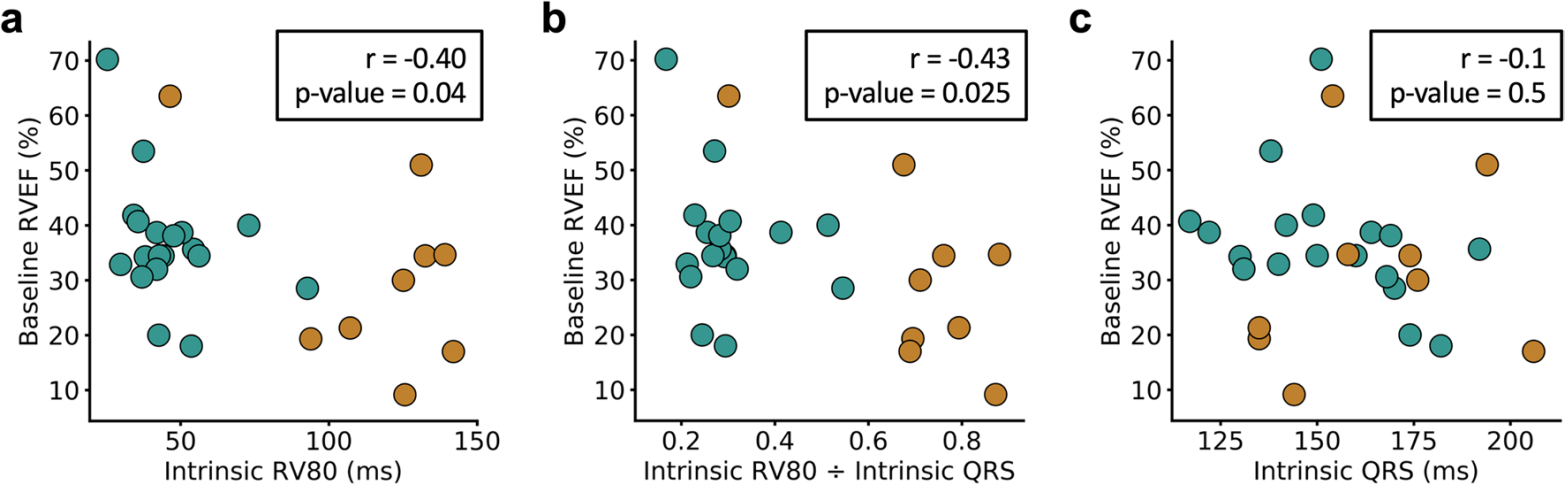
Associations with RV electrical activation and CMR-based RV function. Scatter plots of the baseline RVEF versus the RV80 (**a**), RVEF versus RV80/QRS (**b**), and RVEF versus QRS (**c**) are shown. The strongest associations were found with the RV80 and RV80/QRS duration at baseline

Decreased RVEF pre-CRT was associated with a greater RV80 (more delayed RV electrical activation) pre-CRT overall (*r* =  − 0.40, *p* = 0.04) (Fig. 6a) and a greater RV80/QRS (*r* =  − 0.43, *p* = 0.025) (Fig. 6b). This demonstrates the association between abnormal RV electrical activation patterns and RV dysfunction. We also note that RVEF was generally lower, and both RV80 and RV80/QRS were higher in patients with RBBB compared with LBBB. Of note, one patient with RBBB did not have a delayed RV80 and had better RVEF, which probably represents a case of LBBB masquerading as RBBB [16]. Of note, in contrast to the associations demonstrated with the RV80, there was no association between QRS duration and RVEF (*p* = 0.5) (Fig. 6c), highlighting the value of chamber-specific electrical activation assessment, particularly for the RV.

Supplemental Fig. 2 demonstrates differences in electrical parameters in patients who do and do not meet criteria for specific response endpoints. Specifically, patients with at least a 5% reduction in the LVESV with CRT implemented as synchronized LV pacing had a lower absolute difference between the LV80 and RV80 with that pacing mode. Similarly, patients with at least a 5% reduction in the LVESV with RV pacing had a lower absolute difference between the LV80 and RV80 during RV pacing. In addition, patients with an improvement in the peak VO_2_ of at least 1 mL/kg/minute with biventricular pacing had a more favorable RV80 time with biventricular pacing, highlighting the coupling between exercise capacity and right ventricular function.

## Discussion

In summary, this novel study offers a new paradigm for the application of three-dimensional body surface mapping to CRT by examining the consistency of activation rates of the RV and LV over time, employing activation parameters in the setting of variable RV and LV activation over time, evaluating the impact of cardiac pacing strategies on both parameters and overall clinical response, and assessing three-dimensional electrical activation parameters with multiple dimensions of CRT response. The main finding of this study is that RV-specific or LV-specific assessments of RV and LV activation after CRT have greater associations with CRT response outcomes than biventricular measures of electrical activation derived from the surface electrocardiogram (QRS duration or change in QRS duration result from CRT). These outcomes included improvement in LV systolic function, improvement in exercise capacity based on the peak VO_2_, and the BNP after CRT, which has been of particular interest in several CRT studies [9, 24, 25]. The rationale for demonstrating the association of the electrical findings with these different response findings is that, as stated in the recent 2023 HRS/APHRS/LAHRS guideline statement on cardiac physiologic pacing, CRT response has a “variable definition,” including “improvements in mortality and HF hospitalization, […] improvement in clinical parameters of HF, stabilization of ventricular function, or prevention of progression of HF” [26]. This is consistent with the many different response endpoints reported in the original clinical trials leading to the current CRT indications [27].

In addition, important findings were demonstrated for body surface mapping in the setting of RV dysfunction, delayed RV electrical activation, and RBBB: (1) a subgroup of patients with RBBB who had favorable LV remodeling with RV pacing only was identified; (2) decreased RV function by CMR was shown to be associated with delayed RV electrical activation using body surface mapping but not with the QRSd from the surface electrocardiogram; and (3) RV electrical activation timing was associated with improvement in myocardial oxygen uptake (peak VO_2_). The observations also highlight the role of body surface mapping to assess RV electrical activation, define the pacing strategy in RBBB, and predict the functional response to biventricular pacing.

These RV and LV parameters were constructed as the time to 80% activation of the respective chamber, which makes the parameters less sensitive to long tails at the end of the activation curves. The intuition behind this approach is based on the hypothesis that the time to activation of most of the surface area of the chamber myocardium is more important than the time of activation of the entire ventricular chamber. In other words, the time to 80% activation of the ventricular chamber surface area is considered more important than the time to activation of every ventricular myocyte. The variable rates of RV and LV activation, manifested in the chamber activation versus time function with features such as long tails at the end of the temporal activation curve, was the most prominent in the RV for patients with a LBBB QRS morphology at baseline and the LV for patients with a RBBB QRS morphology at baseline.

The variable coupling of response parameters to LV function to measures of exercise capacity such as the peak VO_2_ has been linked to RV function in the heart failure literature [28]. In our prior work, we have demonstrated the utility of multivariable statistics for analysis of a multidimensional CRT response vector including fractional change in the LVESVI, change in peak VO_2_, and post-CRT BNP [11, 29], as CRT response is complex, and patients may have nonuniform findings with respect to these response parameters. The findings in this paper add to our understanding of how RV electrical and mechanical findings contribute to coupling between LV systolic function and peak VO_2_. In particular, we found that our RV electrical activation parameter was the best predictor of the change in peak VO_2_ after CRT, and RV function pre-CRT by CMR was associated with prolonged RV electrical activation, as parameterized by the RV80. These findings offer important insights into the complexity of CRT response and its associations with RV function and RV electrical activation pre-CRT.

Another important application of this work is the use of three-dimensional electrical mapping to guide the CRT pacing strategy. In particular, we have shown that both left ventricular and biventricular pacing decrease the variability of RV and LV chamber activation to varying degrees. In addition, we have shown the association of a novel electrical assessment of interventricular electrical synchrony (absolute value of the difference in LV80 and RV80) with LV functional improvement after synchronized LVP, the LV80 during biventricular pacing with BNP after CRT, and the avoidance of RV80 prolongation post-CRT with improvements in peak VO_2_.

While we recognize the three-dimensional body surface electrical mapping will likely be a specialized treatment for selected CRT implants in the future, we offer a novel approach for its application to CRT and have shown its utility in specific cases for explaining different manifestations of CRT response, including coupling of LV functional improvement to improvements in exercise capacity and neurohormonal activation. We have also shown the utility of the analysis for predicting response measures based on the resynchronization pacing strategy employed, specifically biventricular or synchronized left ventricular pacing. In addition to advancing the field by demonstrating key mechanisms of CRT response relative to patterns of chamber-specific ventricular activation, we believe that these findings also demonstrate that three-dimensional body surface electrical mapping can have an important clinical impact for many patients with traditional CRT implants. Future applications in patients with conduction system pacing are also of great interest [30].

As a comparison with other approaches to electrical mapping, we contrast the approach in this paper with the vectorcardiography-derived index [31]. The modern vectorcardiography system employs a set of three orthogonal surface leads, one in the right to left direction (x lead), one in the head to foot direction (y lead), and one in the front to back direction (z lead). While vectorcardiography is certainly an interesting approach that can provide evaluation of dyssynchrony, the 3-D electrical mapping approach has several advantages relative to vectorcardiography, including the incorporation of the patients 3-D anatomy from a CT, calculation of RV-specific, LV-specific, and biventricular-specific activation times, and visualization of electrical activation sequences on a 3-D contour of the heart.

## Conclusion

In conclusion, this study demonstrates that 3-D electrical mapping predicted favorable post-CRT outcomes and informed effective pacing strategies. Specifically, the study shows that interventricular electrical dyssynchrony based on the absolute value difference in the LV80 and RV80 predicts LV functional improvement with synchronized LVP, and this same measure during RV pacing demonstrates when RV pacing is expected yield similar results to LVP and BIVP. The RV80 was shown to correlate well with pre-CRT RV function on CMR, and the fractional change in this parameter predicted the greatest increases in functional capacity with BIVP. Lastly, LV80 also predicted lower neurohormone levels with BIVP.

### Limitations

We acknowledge that this was a pilot study with relatively small numbers of patients enrolled; however, the demonstration of statistically significant and reproducible findings in this cohort with respect to CRT mechanisms is impressive and justifies studies in larger cohorts. The study design may be considered both a strength and limitation. The assessment of the acute effects of CRT pacing modes at the follow-up visits over 6 months offers links between the acute mechanical effects of resynchronization with different pacing modes and electrical parameters. While many of the patients had been programmed to the CRT pacing mode studies (BIVP or synchronized LVP) at the time of echocardiographic post-CRT functional assessments, others may have been programmed to BIVP for the past six months, such that the mechanical observations would then be interpreted as more acute effects. Future studies could be designed to also focus on the effects of the pacing modes over longer periods of time as facilitated by larger numbers of patients. Additionally, electrical activation of the septum was not assessed during any pacing mode but may also be useful in predicting CRT outcome.

### Supplementary Information

ESM 1 (TIFF 65004 kb)

High resolution image (PNG 525 kb)

ESM 2 (TIF 709 kb)

High resolution image (PNG 262 kb)

ESM 3 (DOCX 14 kb)

## Abbreviations

CRT: Cardiac resynchronization therapy
LBBB: Left bundle branch block
RBBB: Right bundle branch block
RV: Right ventricle
LV: Left ventricle
QRSd: QRS duration
LV80: Time to 80% of LV activation
RV80: Time to 80% of RV activation
BIV80: Time to 80% of both chambers activation
LVP: Left ventricular pacing
RVP: Right ventricular pacing
BIVP: Biventricular ventricular pacing
LVESVI: Left ventricular end-systolic volume index
BNP: B-type natriuretic peptide
