# Supplementary figures and images for: Noninvasive Electrical Mapping Compared with the Paced QRS Complex for Optimizing CRT Programmed Settings and Predicting Multidimensional Response
Source: J Cardiovasc Transl Res. 2023 Sep 6;16(6):1448–60. doi: 10.1007/s12265-023-10418-1 (PMC10721664; doi:10.1007/s12265-023-10418-1)

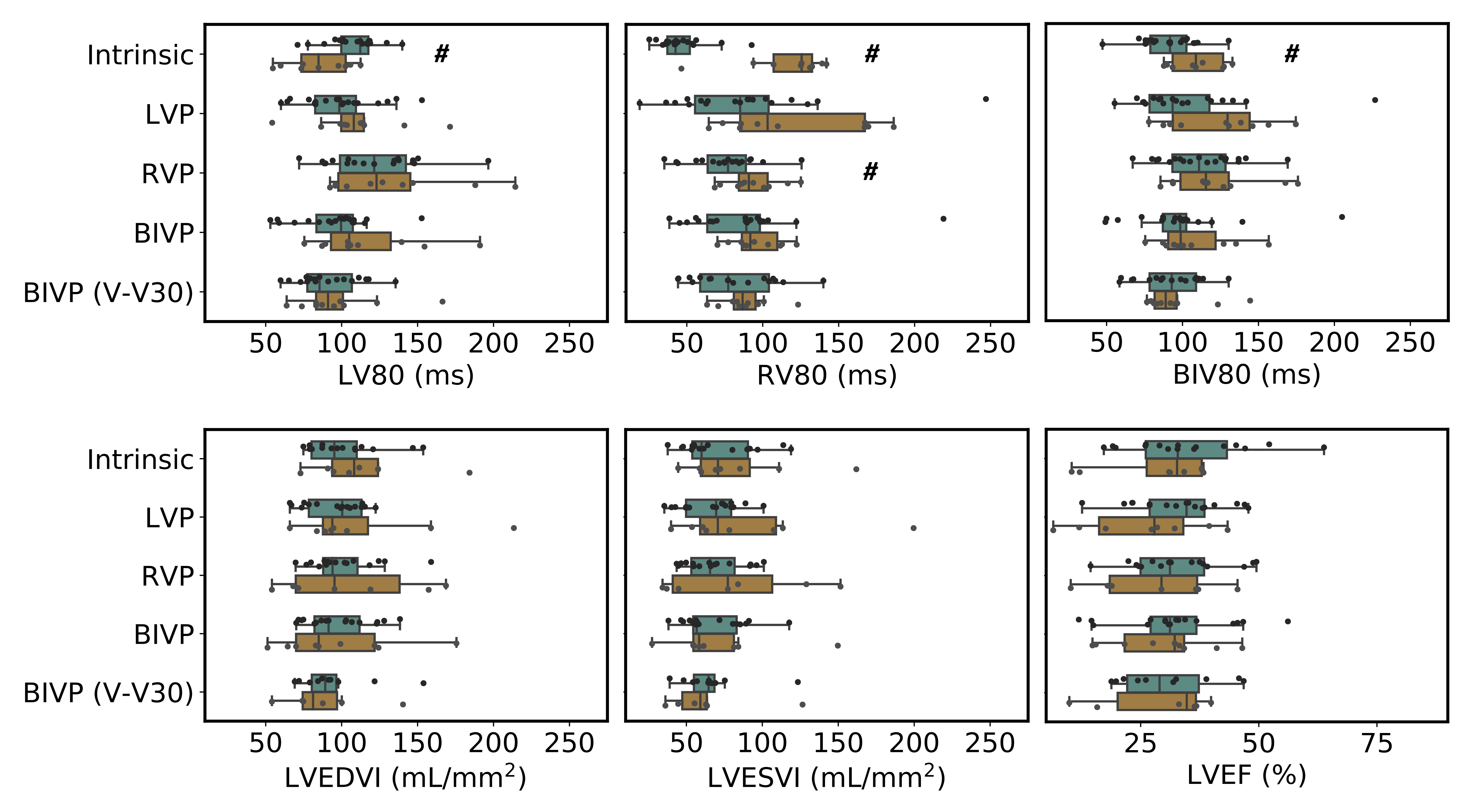

Supplement: Supplementary file 1 — (TIFF 65004 kb) [file 12265_2023_10418_MOESM1_ESM.tiff]

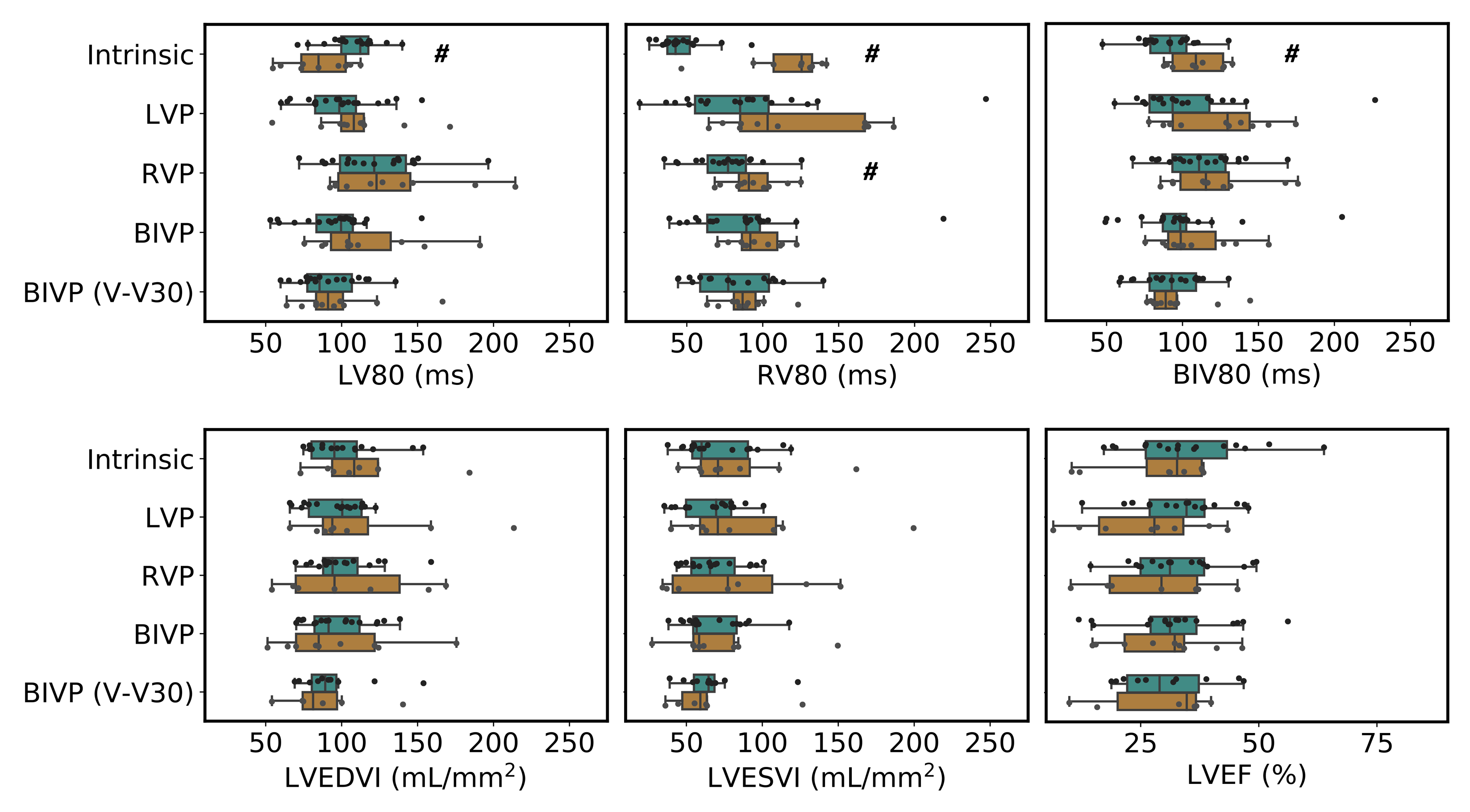

Supplement: Supplementary file 2 — High resolution image (PNG 525 kb) [file 12265_2023_10418_Fig7_ESM.png]

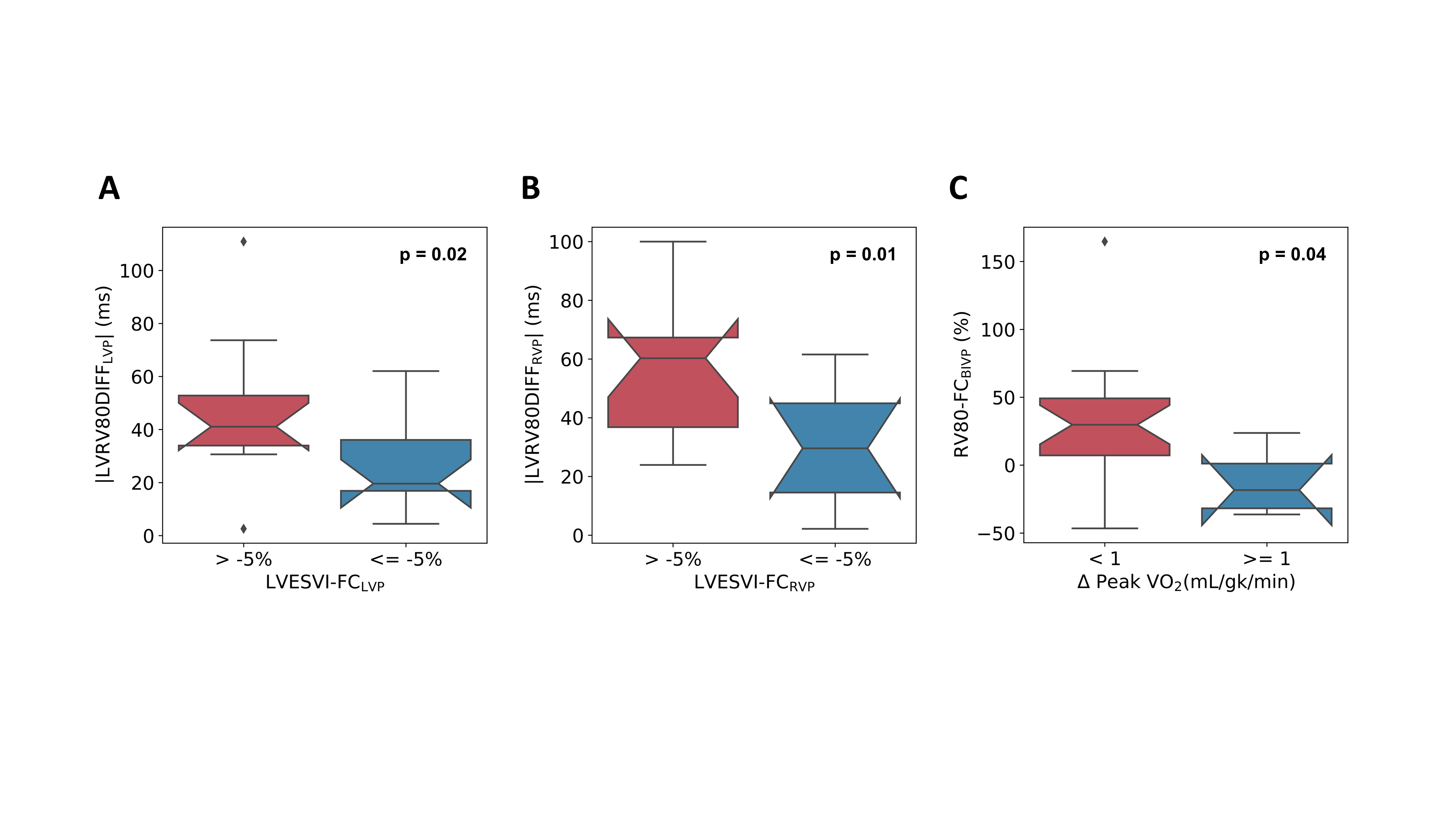

Supplement: Supplementary file 3 — (TIF 709 kb) [file 12265_2023_10418_MOESM2_ESM.tif]

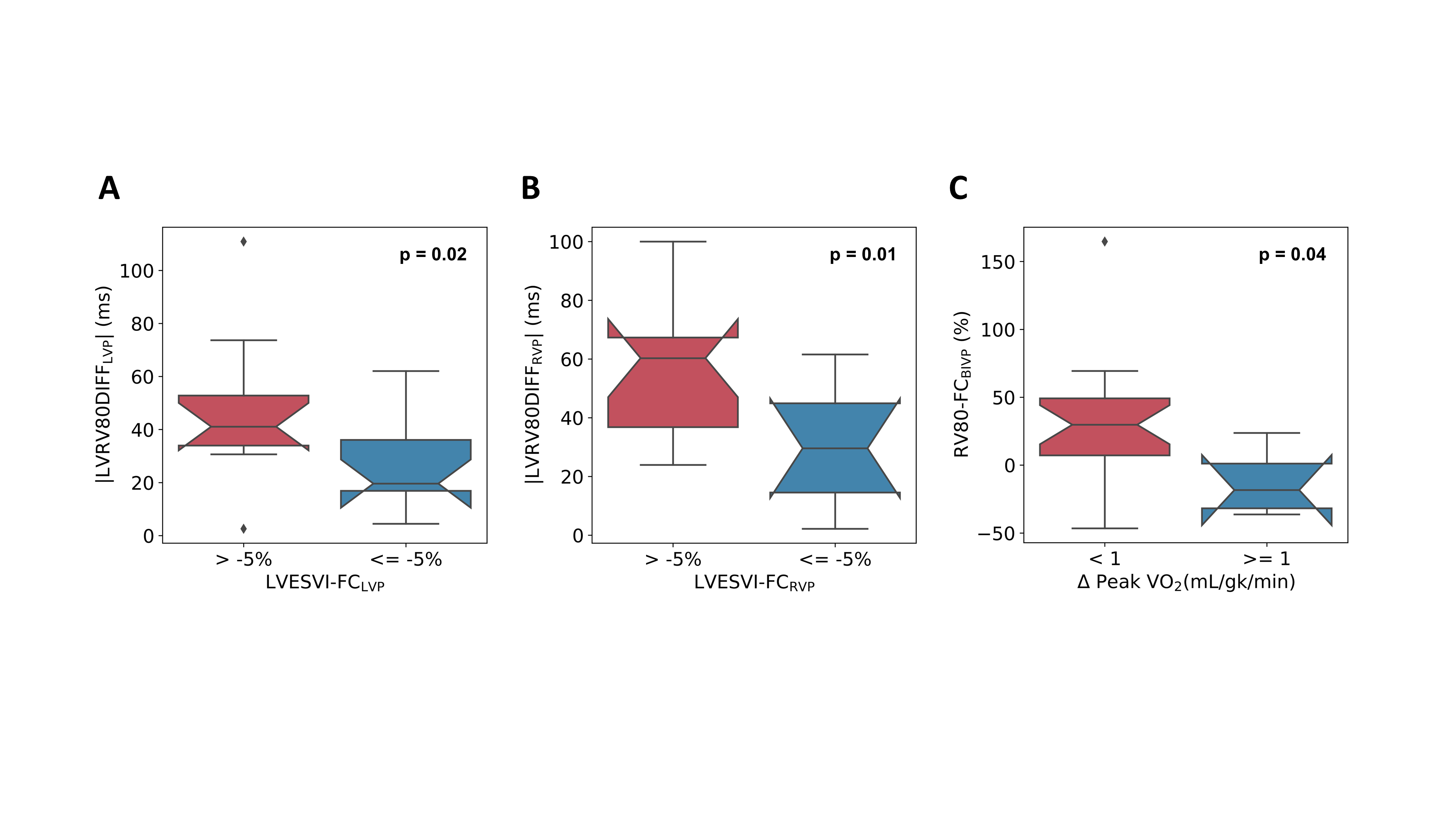

Supplement: Supplementary file 4 — High resolution image (PNG 262 kb) [file 12265_2023_10418_Fig8_ESM.png]
