# Supplementary material for: Noninvasive Electrical Mapping Compared with the Paced QRS Complex for Optimizing CRT Programmed Settings and Predicting Multidimensional Response
Source: J Cardiovasc Transl Res. 2023 Sep 6;16(6):1448–60. doi: 10.1007/s12265-023-10418-1 (PMC10721664; doi:10.1007/s12265-023-10418-1)
Supplement: Supplementary file 5 — (DOCX 14 kb) [file 12265_2023_10418_MOESM3_ESM.docx]

| **Supplemental Table – Normality Check (Continuous Variables)** | | |
| --- | --- | --- |
|  | **Shapiro-Wilk Test Statistic** | **p Value** |
| **Demographics** |  |  |
| Age, years | 0.98 | 0.76 |
| BMI | 0.96 | 0.36 |
| Weight, kg | 0.98 | 0.86 |
| SHFMM | 0.91 | 0.011* |
| **Laboratory Studies, Vital Signs & Exercise Testing** |  |  |
| Systolic BP, mm Hg | 0.94 | 0.094 |
| Sodium, mEq/L | 0.92 | 0.022* |
| Creatinine, mg/dL | 0.91 | 0.017* |
| Hemoglobin, g/dL | 0.98 | 0.70 |
| GFR, mL/min/1.72m^2^ | 0.970 | 0.50 |
| Log(BNP) | 0.97 | 0.67 |
| Peak VO_2_, mL/kg/min | 0.85 | 0.00043* |
| **CMR & Echocardiography Assessment Parameters** |  |  |
| LVEF % | 0.97 | 0.42 |
| LVEDVI mL/m^2^ | 0.85 | 0.00052* |
| LVESVI, mL/m^2^ | 0.85 | 0.00038* |
| RVEF % | 0.92 | 0.024* |
| RVEDVI mL/m^2^ | 0.87 | 0.0011* |
| RVESVI mL/m^2^ | 0.86 | 0.00055* |
| CURE-SVD | 0.97 | 0.58 |
| **Electrical Parameters** |  |  |
| QRS, ms | 0.97 | 0.49 |
| QLV, ms | 0.96 | 0.21 |
| **Response Measures at 6-Months Post-CRT** |  |  |
| Fractional Change in LVESVI | 0.97 | 0.39 |
| Log(BNP) | 0.96 | 0.36 |
| Change in Peak VO_2_, mL/kg/min | 0.88 | 0.0016* |
